# Re-irradiation of Recurrent Pineal Germ Cell Tumors with Radiosurgery: Report of Two Cases and Review of Literature

**DOI:** 10.7759/cureus.585

**Published:** 2016-04-25

**Authors:** Kenneth Wong, Anthony B Opimo, Arthur J Olch, Sean All, Jonathan F Waxer, Desirae Clark, Justine Cheng, Alisha Chlebik, Anat Erdreich-Epstein, Mark D Krieger, Benita Tamrazi, Girish Dhall, Jonathan L Finlay, Eric L Chang

**Affiliations:** 1 Department of Radiation Oncology, Keck School of Medicine of the University of Southern California, Los Angeles, CA; 2 Department of Radiation Oncology, UCLA, Los Angeles, CA; 3 College of Medicine, University of Central Florida College of Medicine, Orlando, FL; 4 School of Medicine, Tulane University School of Medicine, New Orleans, LA; 5 Radiation Oncology Program, Children’s Center for Cancer and Blood Diseases, Children’s Hospital Los Angeles, Los Angeles, CA; 6 Mechanical Engineering Department, Massachusetts Institute of Technology, Cambridge, MA; 7 Neuro-Oncology Program, Children’s Center for Cancer and Blood Diseases, Children’s Hospital Los Angeles, Los Angeles, CA; 8 Department of Pediatrics, Keck School of Medicine of the University of Southern California, Los Angeles, CA; 9 Department of Neurosurgery, Keck School of Medicine of the University of Southern California, Los Angeles, CA; 10 Department of Radiology, Keck School of Medicine of the University of Southern California, Los Angeles, CA; 11 Pediatric Neuro-Oncology, Keck School of Medicine of the University of Southern California, Los Angeles, CA; 12 Pediatric Neuro-Oncology, The Ohio State University, Nationwide Children's Hospital

**Keywords:** Stereotactic Radiosurgery, frameless stereotactic radiotherapy, radiation oncology, gamma knife, linac, head immobilization, cns germ cell tumor, re-irradiation

## Abstract

Primary intracranial germ cell tumors are rare, representing less than 5% of all central nervous system tumors. Overall, the majority of germ cell tumors are germinomas and approximately one-third are non-germinomatous germ cell tumors (NGGCT), which include teratoma, embryonal carcinoma, yolk sac tumor (endodermal sinus tumor), choriocarcinoma, or mixed malignant germ cell tumor. Germ cell tumors may secrete detectable levels of proteins into the blood and/or cerebrospinal fluid, and these proteins can be used for diagnostic purposes or to monitor tumor recurrence. Germinomas have long been known to be highly curable with radiation therapy alone. However, many late effects of whole brain or craniospinal irradiation have been well documented. Strategies have been developed to reduce the dose and volume of radiation therapy, often in combination with chemotherapy. In contrast, patients with NGGCT have a poorer prognosis, with about 60% cured with multimodality chemoradiation. There are no standard approaches for relapsed germ cell tumors. Options may be limited by prior treatment. Radiation therapy has been utilized alone or in combination with chemotherapy or high-dose chemotherapy and transplant. We discuss two cases and review options for frameless radiosurgery or fractionated radiotherapy.

## Introduction and background

Primary intracranial germ cell tumors (IGT) are rare, representing less than 5% of all central nervous system tumors in Western series [1-2] but may be more common in East Asia [3-4]. These tumors most commonly occur in the suprasellar cistern and pineal gland and have a male predominance. Overall, the majority of germ cell tumors are germinomas and approximately one-third are non-germinomatous germ cell tumors (NGGCT), which include teratoma, embryonal carcinoma, yolk sac tumor (endodermal sinus tumor), choriocarcinoma, or mixed malignant germ cell tumor. Embryonal or endodermal sinus tumors are more common in adolescence and young adulthood [3]. Germ cell tumors may secrete detectable levels of proteins into the blood and/or cerebrospinal fluid (CSF), and beta-human chorionic gonadotropin (HCG) and alpha-fetoprotein (AFP) are used for diagnostic purposes and monitor tumor recurrence. Pure germinomas may have elevated HCG [5]. Elevated serum or CSF HCG > 50 mIU/mL and/or elevated AFP are generally considered consistent with NGGCT and biopsy is not required.

Germinomas have long been known to be highly curable with radiation therapy (RT) alone. However, the late effects of whole brain or craniospinal irradiation (CSI) have been well documented, with adverse impacts on hearing, endocrine regulation, neurocognitive function, and risk of secondary malignancies [6-8]. To mitigate these risks, strategies have been developed to reduce the dose and volume of radiation therapy, often in combination with chemotherapy. In contrast, only about 20-45% of patients with NGGCT can be cured following radiation therapy alone, though results are improved to about 60% with multimodality chemoradiation [1].

The focus of this paper is to discuss treatment options for locally relapsed IGT without dissemination and to investigate patient and/or tumor characteristics that may affect the choice of re-irradiation modalities, such as stereotactic radiosurgery (SRS), hypofractionated fractionated stereotactic radiotherapy (FSRT), or full dose re-irradiation with external beam RT.

### Case reports

Case 1

**Figure 1:**
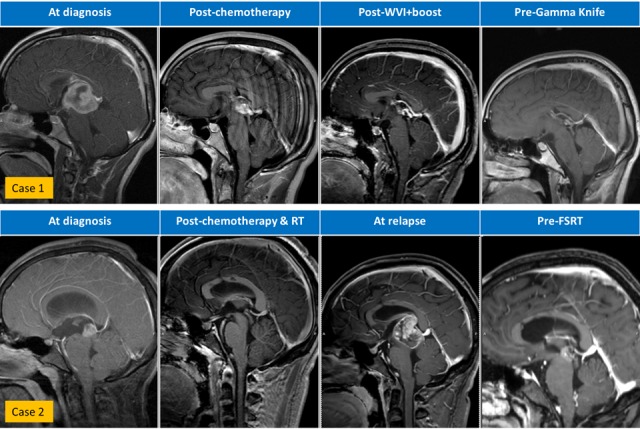
Serial sagittal T1-weighted MRI brain scans with gadolinium WVI = whole ventricular irradiation; CSI = craniospinal irradiation; FSRT = fractionated stereotactic radiotherapy; RT = radiotherapy

**Figure 2:**
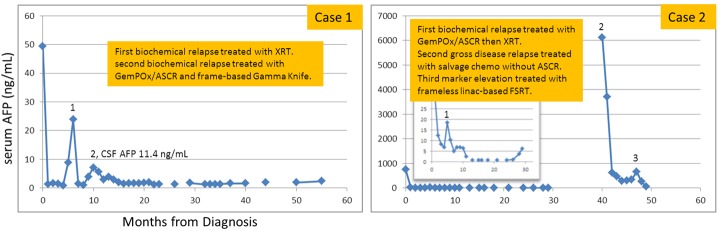
Serial values for serum AFP

**Figure 3:**
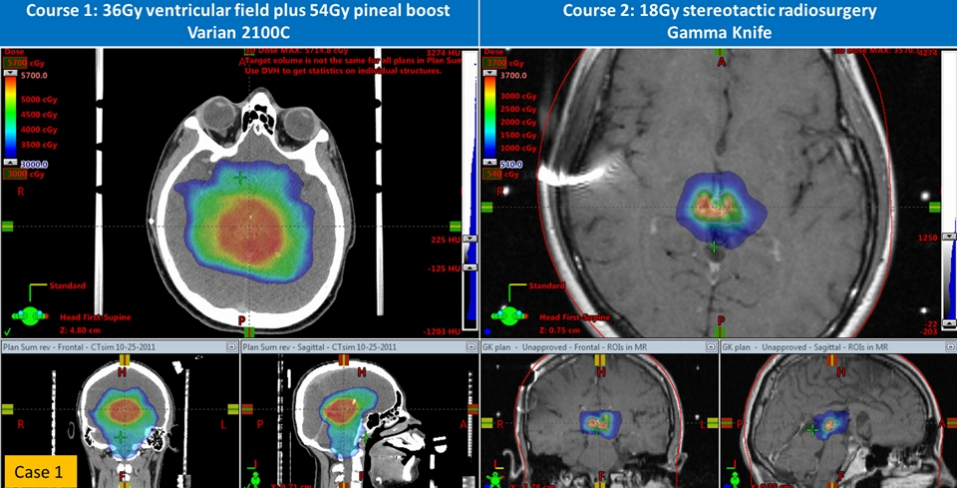
Comparison of initial and re-irradiation treatment plans (axial, coronal, and sagittal images) for Case 1

A 16-year-old Hispanic male without prior health problems presented with gradual memory loss and severe headache; an MRI brain with gadolinium revealed an enhancing 3.5 x 3.4 x 3.7 cm pineal gland tumor (Figure 1). His serum AFP was 49.3 ng/mL and CSF AFP was 33.9 ng/mL (Figure 2). Both serum and CSF HCG were negative. An MRI spine and CSF cytology were negative. He had hydrocephalus and an intratumoral hemorrhage following a ventriculostomy and ventriculoperitoneal (VP) shunt placement (Table 1). His neurological status deteriorated and he became unresponsive. Because of his intratumoral bleed and performance status, he was treated with systemic chemotherapy as per the Children’s Oncology Group (COG) Trial ACSN0122 with alternating carboplatin/etoposide and ifosfamide/etoposide. Following his first cycle of chemotherapy, he began to neurologically recover and his tumor markers normalized after two cycles of chemotherapy. After six cycles of chemotherapy, his serum and CSF tumor markers remained undetectable with a residual 1.3 x 2.1 x 1.3 cm enhancing pineal gland mass. About six weeks post-chemotherapy and before RT, his serum AFP rose to 8.9 ng/mL (institutional high normal: 7.3 ng/mL). MRI of the spine was negative. Although concerned about relapse, we began whole ventricular irradiation (WVI) and intensity-modulated radiation therapy (IMRT) with an intended dose of 30.6 Gy (Figure 3). Two weeks after starting WVI, his serum AFP increased to 23.9 ng/mL, and five days later was 15.3 ng/mL. With this AFP elevation, we changed his WVI to 36 Gy and subsequently completed an IMRT boost to the pineal gland to a cumulative total dose of 54 Gy. After peaking at 23.9 ng/mL early during RT, his serum and CSF AFP became undetectable one-month post-RT. His MRI brain showed a continued mild decrease in the size of enhancing residual tissue. Unfortunately, three months after RT, his CSF AFP was elevated at 11.4 ng/mL (serum 5.4 ng/mL), and MRI of the brain showed an interval increase in the size of enhancing tissue of the pineal gland. He was enrolled in a clinical trial of gemcitabine, paclitaxel, and oxaliplatin (GemPOx), and his CSF AFP became undetectable. After three cycles of GemPOx, he proceeded to consolidation chemotherapy with carboplatin, etoposide, and thiotepa, followed by autologous hematopoietic stem cell rescue (ASCR). He tolerated the transplant well and was discharged on Day 20. He was subsequently referred for stereotactic radiosurgery at an adult hospital where he received treatment on Day 97 (Table 2). He was treated with Gamma Knife (Elekta, Stockholm, Sweden) SRS to 18 Gy in one fraction (Figure 3, Table 3). With 34 months of follow-up post-SRS, his tumor markers remain normal with a stable MRI of the brain.

**Table 1:** Clinical Characteristics at Presentation, First and Second Recurrences M = male; sAFP = serum AFP; cAFP = CSF AFP; VP = ventriculoperitoneal; carbo = carboplatin; ifos = ifosfamide; VP-16 = etoposide; chemo = chemotherapy; WVI = whole ventricular irradiation; CR = complete response; RT = radiation therapy; ETV = endoscopic third ventriculostomy; PR = partial response; GemPOx = gemcitabine, paclitaxel, oxaliplatin; ASCR = autologous hematopoietic stem cell rescue; CSI = craniospinal irradiation; RT = radiotherapy

Case	Age / Sex	Histology	Extent of Disease	Tumor Markers	Surgery	Chemo	Chemo Response	Progression	Treatment & Response	Second Recurrence
1	16M	-	Pineal	sAFP 49.3 ng/mL, cAFP 33.9 ng/mL	Tumor bleed and VP shunt	Carbo/VP-16, Ifos/VP-16	CR post-6^th^ cycle	1 month post-chemo, sAFP 8.9 ng/mL	WVI 36 Gy, plus boost to 54 Gy, CR	3 months post-RT, cAFP 11.4 ng/mL
2	17M	Yolk sac 80% and germinoma 20%	Pineal	sAFP 755 ng/mL, cAFP 350 ng/mL	ETV and biopsy	Carbo/VP-16, Ifos/VP-16	PR post-6^th^ cycle	2 months post-chemo, sAFP 17.8 ng/mL	GemPOx with ASCR, PR, sAFP 5.2 ng/mL, then CSI 36 Gy, plus boost to 54 Gy, CR	29 months post-RT, sAFP 6120 ng/mL, and cAFP 3000 ng/mL

**Table 2:** Clinical Characteristics at Second and Third Recurrences FU = Follow up; GemPOx = gemcitabine, paclitaxel, oxaliplatin; ASCR = autologous hematopoietic stem cell rescue; GK SRS = Gamma Knife stereotactic radiosurgery; CR = complete response; ifos = ifosfamide; VP-16 = etoposide; sAFP = serum AFP; PR = partial response; chemo = chemotherapy; FSRT = fractionated stereotactic radiotherapy

Case	Second Recurrence / Extent of Disease	Subsequent Treatment	Response	Third Recurrence	Subsequent Treatment	Response	FU Post-progression
1	Tumor marker elevation	GemPOx with ASCR, GK SRS	CR	-	-	-	Alive, 34 months
2	Pineal gross disease and tumor marker elevation	Cisplatin/Ifos/VP-16, BCNU/VP-16/Cisplatin	PR	1 month post-chemo, sAFP 490 ng/mL	FSRT followed by oral VP-16 & thalidomide	PR, sAFP 19.8 ng/mL	Alive, 3 months

**Table 3:** Comparison of Different Radiosurgery Techniques for Case 1 and Case 2 *dose to PTV overlapping brainstem **Indices derived for total PTV (includes volume overlapping brainstem) RT1 = first course of radiotherapy; RT2 = second course of radiotherapy; IMRT = intensity-modulated radiation therapy; SRS = stereotactic radiosurgery

Case	Technique & Interval Between RT1 & RT2	Immobilization	PTV Volume & Prescription Dose	Shots / Beams	Dose Statistics		Conformity	Gradient
1	Gamma Knife RT to SRS: 9.5 months	Head frame	2.4 cm^3^, 18 Gy to 50% isodose line	14 shots	18 Gy margin, 36 Gy max		1.46	2.94
2	Dose painting IMRT RT to FSRT: 36 months	Frameless vacuum-assisted mouthpiece with surface imaging	4.3 cm^3^, 25 Gy to 79.4% with limit of 20 Gy to brainstem	8 beam non-coplanar IMRT	26.4 Gy mean, 31.5 Gy max	19.1 Gy* mean, 22.8 Gy* max	0.59**	0.91**

Case 2

**Figure 4:**
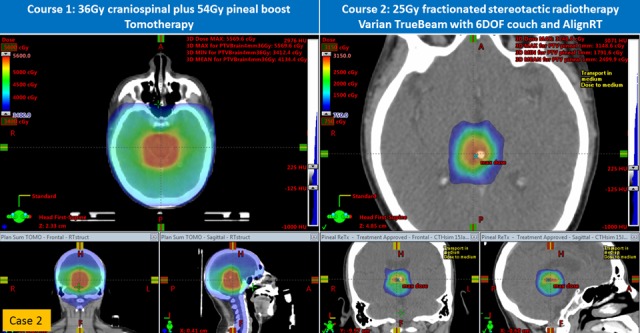
Comparison of initial and re-irradiation treatment plans (axial, coronal, and sagittal images) for Case 2

A 17-year-old Hispanic male without prior health problems presented with headaches and multiple episodes of vomiting. He had an MRI of the brain, which showed a pineal gland tumor with hydrocephalus (Figure 1). Upon transfer to our institution, he had an endoscopic third ventriculostomy and biopsy, which revealed a mixed malignant germ cell tumor (80% yolk sac and 20% germinoma). His serum AFP was 755 ng/mL, a normal serum HCG, and the CSF AFP was 350 ng/mL (CSF HCG: 13). An MRI of the brain revealed a 1.5 x 1.3 x 1.3 cm T1-enhancing pineal region mass. An MRI of the spine and CSF cytology were negative. He was also treated as per COG ACNS0122 with six cycles of chemotherapy. His serum AFP reached a nadir of 7 ng/mL and CSF AFP was 17.4 ng/mL; serum and CSF HCG were negative. MRI of the brain showed only a small residual enhancement in the region of the pineal gland. About six weeks after chemotherapy and before his planned RT, his serum AFP rose to 17.8 ng/mL and the CSF AFP rose to 26.5 ng/mL (see inset graph on Figure 2). Instead of proceeding to RT as in Case 1, our patient was enrolled on the GemPOx clinical trial; after four cycles, his serum and CSF AFP decreased to 4.6 and 5.2 ng/mL, respectively, (Table 1). With post-chemotherapy serum and AFP stable at 5.2 and 6.8 ng/mL, respectively, he proceeded to consolidation chemotherapy with ASCR. After he recovered from the transplant, he started RT with 36 Gy CSI with TomoTherapy® (Accuray, Inc., Sunnyvale, CA) followed by an IMRT boost to the pineal gland for a cumulative dose of 54 Gy (Figure 4). One month post-RT, his serum and CSF AFP became undetectable. He was then followed for 16.5 months, after which he was lost to follow-up. He returned almost one year later with morning headaches and an MRI of the brain showed a large partially hemorrhagic, enhancing pineal region mass measuring 3.6 x 3.1 x 3.4 cm (Figure 1). He had a markedly elevated serum AFP, and CSF AFP was over 3,000 ng/mL (Figure 2). MRI of the spine was negative for leptomeningeal metastases with negative CSF cytology. He was salvaged with systemic chemotherapy (cisplatin, ifosfamide, etoposide for five cycles with one intervening cycle of BCNU, etoposide, and cisplatin). Initially, his serum AFP rapidly declined with chemotherapy but plateaued with a mean of 268 ng/mL. Because of prior treatment, his hematopoietic cell recovery was prolonged. After the sixth cycle of chemotherapy, his serum AFP rose to 490 ng/mL. At this point, he was considered for re-irradiation with SRS. MRI of the brain demonstrated a residual enhancing mass measuring 1.7 x 1.7 x 1.4 cm intimately associated with the thalamus, tectum, and midbrain. With a history of prior RT and involvement of brainstem and thalamus, we decided to offer fractionated stereotactic radiotherapy rather than single fraction SRS. The patient underwent CT simulation with a vacuum-assisted mouthpiece head immobilization system with 1.5 mm slice spacing and intravenous contrast. A gadolinium-enhanced MRI of the brain with 1 mm spacing was obtained and rigidly registered with the simulation CT scan. The gross target volume (GTV) was defined by a team of radiation oncologists, a neuroradiologist, and a neurosurgeon. A 1 mm margin was added to create the planning target volume. A dose of 25 Gy in five fractions was prescribed with a constraint of 20 Gy to the brainstem (Table 3). Dose-painting IMRT (Figure 4) was planned with the Eclipse treatment planning system, version 13.6 (Varian, Palo Alto, CA), and delivered on a Varian TrueBeam with a PerfectPitch™ 6-DOF (degrees of freedom) couch (Varian, Palo Alto, CA) with kVue couch top (Qfix, Avondale, PA). Cone beam CT (CBCT) daily image guidance was used for alignment to the calcified portion of the residual tumor. Intrafraction real-time optical surface monitoring system (OSMS) was performed with surface imaging using AlignRT (VisionRT, London, UK). The patient tolerated FSRT well with Grade 2 fatigue. At the start of the FSRT, his serum AFP was 656 ng/mL, peaked at 832 ng/mL, and decreased by 40% two weeks after FSRT. Oral etoposide and thalidomide were then added, and 2.5 months post-treatment, the serum AFP fell to 6.9 ng/mL.

## Review

These two cases contribute insight to the series demonstrating that recurrent germ cell tumors can be sensitive to chemotherapy and re-irradiation [9-12]. In the series described by Zissiadis, et al. [9], one patient with NGGCT recurred after subtotal resection, chemotherapy, and CSI. That patient, who subsequently received high-dose chemotherapy with ASCR and 15 Gy SRS, was alive at 32 months post-salvage therapy. Modak, et al. [10] described 21 relapsed IGT patient treated with high-dose chemotherapy and ASCR. There were five survivors among the twelve patients with NGGCT, and of those survivors, two had RT and one was treated focally, but not with SRS. Hasegawa, et al. [11] successfully salvaged a patient with chemotherapy and Gamma Knife SRS. In contrast, chemotherapy alone is not likely to be effective up front [1, 13-15] or at relapse [16], and avoiding RT with high-dose chemotherapy and ASCR is uncertain [10, 12, 17].

Consideration for re-irradiation must take into account size and location of tumor recurrence, prior treatments, the time interval from prior radiation therapy, the proximity of organs-at-risk (OAR), and the need for anesthesia. Not all patients are candidates for single fraction radiosurgery. In cases where re-irradiation has been performed with curative intent for medulloblastoma or ependymoma, brainstem toxicity has been an issue [18-22]. Less toxicity has been described with FSRT or conventional fractionation [23-24], and thus, these may be safer techniques. Effective palliation in children with recurrent or metastatic tumors with frameless SRS or FSRT can be achieved with attention to cumulative doses to critical structures [25]. Similarly, palliation in adults with brainstem metastases with the CyberKnife SRS/FSRT has been described with limited acute brainstem toxicity [26].

Our second case highlights some of the potential advantages of frameless radiosurgery, which include increased patient comfort, ability to fractionate treatment, greater time for the multidisciplinary team review of imaging, contours, and dosimetry, and shorter daily treatment appointments. For children, frame placement may be a higher risk due to their thinner and softer skulls and need for sedation, so frameless FSRT can be more a more acceptable option. When re-irradiation is planned, a diagnostic MRI should be obtained within two weeks of the simulation scan [27].

There are three main types of frameless immobilization: thermoplastic mask, open thermoplastic mask with or without bite block, and upper jaw fixation devices (bite block or vacuum-assisted mouthpieces) [28]. These devices are commonly used in conjunction with custom cushions conformed to the head or head and shoulder. Thermoplastic masks and vacuum-assisted mouthpiece systems seem to have similar accuracy and precision [29-31], although masks tend to be less rigid. In addition, some investigators have found the mask to be more comfortable [29]. However, in our experience over the past decade, children by far chose the mouthpiece system over a closed thermoplastic mask, which was described as ”scary” and “too tight.” We have used the vacuum-assisted mouthpiece with high accuracy in infants or edentulous patients [32].

Treatments can be planned with cylindrical collimators, dynamic conformal arcs, 3D conformal beams, IMRT (step-and-shoot or sliding window, coplanar or non-coplanar), volumetric modulated arc therapy (VMAT), or proton beams [9, 25, 33-38]. With the Extend frameless immobilization system (Elekta, Stockholm, Sweden) [39], fractionated Gamma Knife radiosurgery is possible [40] and is further supported by CBCT in the Icon system (Elekta, Stockholm, Sweden). Case 2 was treated with IMRT in order to reduce the dose to the adjacent brainstem, with IMRT being the best way to achieve dose-painting for a simultaneous integrated boost.

**Figure 5:**
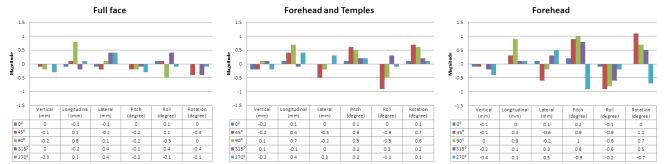
Phantom study demonstrating increased variability of OSMS-reported 6-DOF couch shifts as the region of interest size decreases at five couch angles OSMS = Optical Surface Monitoring System; DOF = degrees of freedom

Treatment delivery can be accomplished on a variety of different platforms with different equipment, including linear accelerators, Gamma Knife, CyberKnife, TomoTherapy, or protons (Table 4). Some consider frameless immobilization systems to be less precise, even though patients can shift within frames and most frame-based systems ignore rotational shifts. To address this concern, orthogonal or stereoscopic kilovoltage (kV) or CBCT imaging guidance can permit shifts to correct for setup or immobilization inaccuracies. The time required for image guidance (acquisition, review, and adjustment) in the second case was a mean of 9 minutes (range: 4-13) and was reasonable and comparable to other investigators [34, 41]. Our workflow was similar to that described by Li, et al. [29]. In some centers, as a proxy for intrafraction motion, post-treatment imaging is often performed. More recently, real-time intrafraction monitoring can be performed with surrogate markers or the body surface and can interrupt treatment when movement exceeds a predefined tolerance (1-2 mm and 1°) [29, 41-42]. At our institution, we conducted a phantom study, which demonstrated the variability of the OSMS when the region of interest was decreased in size and as the couch angle changed (Figure 5). Based on these results, we utilized an intermediate patch monitoring the forehead and temples which were not obscured by the mouthpiece system. Over five treatments, the patient had very small intrafraction shifts (Table 5) while immobilized for a mean of 29 minutes (range: 21-42 min) with a mean treatment time of 19 minutes (range: 12-27 min). Mayo, et al. noted that their treatment times with noncoplanar VMAT were about 20 minutes and shorter than the 45-60 minutes required for frame-based treatment [34].

**Table 4:** Literature Review of Frameless Radiosurgery (Selected Series) Ref = references; Pre-RT = pre-radiation therapy; HD = high definition; TPS = treatment planning system; IR = Infrared camera system with 4-6 reflectors or emitters mounted on bite-block tray; OSMS = optical surface monitoring system (AlignRT); kV = kilovoltage imaging; CBCT = cone beam CT; DOF = degrees of freedom; HD = high definition; MLC = multileaf collimator; SRS = stereotactic radiosurgery; VMAT = volumetric modulated arc therapy; FSRT = fractionated stereotactic radiotherapy; OBI = on-board imaging; GK = Gamma Knife; DCA = dynamic conformal arc; FFF = flattening fillter free; N = number; mets = metastases; OBI = on-board imager; AVM = arteriovenous malformation; CI = conformity index; HI = homogeneity index; GTV = gross target volume; PTV = planning target volume; IMRT = intensity modulate radiation therapy

First Author [Ref], Institution, Publication year	Equipment	Image Guidance, Robotic Couch, Intrafraction Motion	Pre-RT Scans, Immobilization Devices	Patients	Technique, TPS	Notes, Results, or Conclusions
Mancosu [43] Milan-Rozzano 2016	Varian Edge 120HD MLC	kV/CBCT 6-DOF couch OSMS	CT MRI	Phantom	-	Study of Edge linac with OSMS and CBCT. Tested ability of OSMS vs. CBCT ability to detect facial movements at isocenter, ability to recognize shifts, at different couch angles, and accuracy of OSMS when a camera is blocked. Submillimeter accuracy with rotational inaccuracy of 0.3 degrees.
Wen [44] Henry Ford 2015	Varian Edge 120HD MLC	kV/CBCT 6-DOF couch OSMS	-	Commissioning	FFF VMAT Cones	Report of commissioning of Edge radiosurgery system. Deviation between OSMS and CBCT was -0.4, 0.1, and 0 mm in vertical, longitudinal, and lateral dimensions. Beam data and mechanical parameters similar to TrueBeam, with advanced imaging package, 6-DOF couch, and intracranial SRS accessory package.
Seravalli [45] MAASTRO 2015	Elekta Synergy 10 mm MLC	kV/CBCT Pre- & post- CBCT	CT 1.2 mm MRI 1.2 mm Mask (BlueBAG)	N = 52 Brain mets	SRS Coplanar VMAT (Pinnacle)	Process of treatment. End-to-end test. GTV-PTV margin of 2.4 - 3.1 mm. Used Quantec constraints.
Li [29] MSK 2015	Varian Trilogy kV/CBCT	OSMS	Bite block (PinPoint) vs Open Mask (Freedom)	N = 25 Bite block N = 8 Mask	FSRT Coplanar beams (iPlan)	Process of care diagram. Deliberate forced moves (15 volunteers) on ref Table 1. Study of volunteer comfort ref Table 2.
McTyre [40] Wake Forest 2015	Gamma Knife Perfexion	No OBI	CT MRI Bite block (Extend)	N = 34	Fractionated GK (GammaPlan)	Meningioma, schwannoma, metastases. GTV was treated without margin. 16-32 Gy to 50% isodose line over 4-5 fractions. Optic apparatus constrained to 4 Gy tangential to tumor. Daily repositioning errors < 1.2 mm.
Nanda [25] Emory 2014	Novalis Tx HD MLC	kV/CBCT IR 6-DOF	CT 0.625 mm MRI	N = 5 Pediatric	SRS/FSRT Non-coplanar DCA IMRT 12 beams	GTV-PTV 1 mm 4/5 patients required anesthesia
Pan [41] UCSD 2012	TrueBeam Trilogy	OSMS	CT 1.25 mm MRI 1.25 mm Open Mask (CIVCO)	N = 44 Adults	SRS/FSRT Multiple beams Cones or VMAT (Eclipse)	GTV-PTV 1 mm. Beam hold 1-2 mm and 1°. Treatment times – CBCT mean 11 min. Median shifts 1 mm, 2 mm, 1 mm vertical, longitudinal, lateral. Treatment time 15 min (shorter for TrueBeam). Compared local control to other series.
Schlesinger [39] UVA 2012	Gamma Knife Perfexion	No OBI	CT MRI Bite block (Extend)	N = first 10	Fractionated GK (GammaPlan)	Interfraction and intrafraction performance of Extend. Mean radial setup difference was 0.64 mm, SD 0.24 mm. Mean intrafractional positional difference was 0.47 mm. Cannot account for rotations.
Lu [35] BIDMC 2012	Proton	Orthogonal kV Three 2 mm gold fiducial spheres	CT Frameless	N = 1 AVM	Proton	Description of novel technique with implanted fiducials to localize AVM identified on angiography and to transfer location information to CT for proton SRS planning.
Tryggestad [30] JHU 2011	Elekta Synergy S	Pre- & post- CBCT	Mask - 4 types Nonrandom study Retrospective	N = 121	FSRT/external RT	Demonstrated masks (ref. Figure 1). Best was type-S head and shoulder mask with head and shoulder cushion with mouthpiece. Can achieve intrafraction motion of 1 mm or less, and interfraction variability of less than 3 mm.
Ramakrishna [31] DFCI 2010	Novalis	Stereoscopic kV (ExacTrac) IR	Frame (Radionics) Mask (BrainLAB)	N = 102 SRS N = 7 FSRT	SRS	End-to-end overall accuracy of Novalis Body ExacTrac is 0.7 mm ± 0.3 mm. Approximately 22% of mask-immobilized patients displayed intrafraction displacement of 1-2 mm.
Peng [49] UF Gainesville 2010	Elekta Synergy Varian Trilogy		CT 2 mm Mask IR CBCT	N = 15 IR N = 18 Mask	-	Comparison of IR tracking system setup with CBCT. Setup with IR resulted in setup errors of 1.2 mm determined by CBCT, versus mask and laser setup errors of 3.2 mm. FSRT should not rely on IR alone.
Mayo [34] U Mass 2010	Varian Trilogy 5 mm MLC	kV/CBCT	CT 1.25 mm MRI 1.25 mm Mask (Alpha Cradle)	N = 12 Adults Brain mets	SRS Non-coplanar VMAT (Eclipse)	GTV-PTV 1-2 mm margin. Dosimetric details compared to CyberKnife, TomoTherapy, & IMRT. Reported on CI, gradient, & HI. Phantom end-to-end testing. Compared dose rate vs. survival in cell line (ref Figure 9).
Keshavarzi [50] UCSD 2009	Varian Trilogy	IR	CT 1.25 mm MRI 1.5 mm Mask (AccuForm)	N = 12 Pediatric	SRS/FSRT MLC IMRT Cones (Eclipse)	GTV-PTV margin 1-3 mm

**Table 5:** Six Degrees of Freedom Couch Shifts Based on Daily Image Guidance with CBCT and OSMS *Representative real-time delta shifts across non-coplanar treatment couch angles OSMS = optical surface monitoring system; CBCT = cone beam CT; FSRT = fractionated stereotactic radiotherapy

	Mean Translational Shifts (mm)			Mean Rotational Shifts (°)		
	Vertical	Longitudinal	Lateral	Pitch	Roll	Rotation
Localization CBCT	3.1 (1.7 to 5.4)	0.34 (-0.4 to 1.3)	1.0 (0.5 to 1.5)	0.4 (0.1 to 0.7)	0.1 (0 to 0.2)	-0.1 (-0.3 to 0.2)
Setup OSMS	5.4	-0.40	0.96	0.07	-0.08	0.18
Verification CBCT	0	0.15	-0.3	0.05	0.05	0
Intrafraction OSMS*	0.30	-0.29	0.02	0	-0.01	0.07
Post-FSRT CBCT	-0.2	0.85	-0.25	0.2	0.05	0.15

Some common features of the latest equipment for radiosurgery include: higher mechanical precision, higher dose rate, smaller collimators, image guidance, intrafraction motion detection, and robotic 6DOF couches. Several investigators have performed end-to-end accuracy tests [43-44] and have found the equipment to be highly accurate and suitable for frameless SRS, with GTV-PTV margins of 1-2 mm [31, 34, 41]. By comparison, an end-to-end test with older equipment utilizing 10 mm MLC leaves without 6-DOF couch advocated a GTV-PTV margin of 2.8 mm [45].

## Conclusions

Overall, intracranial germ cell tumors are rare. There are no standard approaches for patients with recurrent germ cell tumors. Curative options are limited by prior treatment. For patients with pure germinomas treated initially with either radiation or chemotherapy [36, 46], high salvage rates are achieved. However, for patients with prior chemoradiation or those with relapsed NGGCT, sustained responses to commonly used salvage chemotherapy regimens are difficult to achieve. To date, cure rates of about 50% have been achieved using a salvage paradigm with an initial intensive chemotherapy to achieve minimal residual tumor, followed by high-dose chemotherapy with ASCR. However, compared to germinomas, relapsed NGGCT patients have a worse prognosis with two-thirds progressing within 18 months of treatment.

When re-irradiating recurrent IGT, the cumulative dose to the optic apparatus or brainstem will often be an issue since these tumors tend to occur in the suprasellar cistern or pineal gland. Data from re-irradiation of pediatric posterior fossa tumors or radiosurgery of lesions near critical structures can inform us about radiobiological dose constraints and guide treatment planning [47-48]. Fractionated treatments may have a lower risk of toxicity.

Frameless immobilization is the best choice for multiple repeated treatments. With our current technology and policies and procedures, we can safely and accurately deliver either SRS or FSRT. With short follow-up, decrement in the tumor markers in our second patient indicated a partial response, although further follow-up is needed to assess response and toxicity.

## References

[1] Kortmann RD (2014). Current concepts and future strategies in the management of intracranial germinoma. Expert Rev Anticancer Ther.

[2] Echevarría ME, Fangusaro J, Goldman S (2008). Pediatric central nervous system germ cell tumors: a review. Oncologist.

[3] Jennings MT, Gelman R, Hochberg F (1985). Intracranial germ-cell tumors: natural history and pathogenesis. J Neurosurg.

[4] McCarthy BJ, Shibui S, Kayama T, Miyaoka E, Narita Y, Murakami M, Matsuda A, Matsuda T, Sobue T, Palis BE, Dolecek TA, Kruchko C, Engelhard HH, Villano JL (2012). Primary CNS germ cell tumors in Japan and the United States: an analysis of 4 tumor registries. Neuro Oncol.

[5] Matsutani M; Japanese Pediatric Brain Tumor Study Group (2001). Combined chemotherapy and radiation therapy for CNS germ cell tumors--the Japanese experience. J Neurooncol.

[6] Jinguji S, Yoshimura J, Nishiyama K, Aoki H, Nagasaki K, Natsumeda M, Yoneoka Y, Fukuda M, Fujii Y (2013). Factors affecting functional outcomes in long-term survivors of intracranial germinomas: a 20-year experience in a single institution. J Neurosurg Pediatr.

[7] van Dijk IW, Cardous-Ubbink MC, van der Pal HJ, Heinen RC, van Leeuwen FE, Oldenburger F, van Os RM, Ronckers CM, Schouten-van Meeteren AY, Caron HN, Koning CC, Kremer LC (2013). Dose-effect relationships for adverse events after cranial radiation therapy in long-term childhood cancer survivors. Int J Radiat Oncol Biol Phys.

[8] Armstrong GT, Liu Q, Yasui Y, Huang S, Ness KK, Leisenring W, Hudson MM, Donaldson SS, King AA, Stovall M, Krull KR, Robison LL, Packer RJ (2009). Long-term outcomes among adult survivors of childhood central nervous system malignancies in the Childhood Cancer Survivor Study. J Natl Cancer Inst.

[9] Zissiadis Y, Dutton S, Kieran M, Goumnerova L, Scott RM, Kooy HM, Tarbell NJ (2001). Stereotactic radiotherapy for pediatric intracranial germ cell tumors. Int J Radiat Oncol Biol Phys.

[10] Modak S, Gardner S, Dunkel IJ, Balmaceda C, Rosenblum MK, Miller DC, Halpern S, Finlay JL (2004). Thiotepa-based high-dose chemotherapy with autologous stem-cell rescue in patients with recurrent or progressive CNS germ cell tumors. J Clin Oncol.

[11] Hasegawa T, Kondziolka D, Hadjipanayis CG, Flickinger JC, Lunsford LD (2003). Stereotactic radiosurgery for CNS nongerminomatous germ cell tumors. Report of four cases. Pediatr Neurosurg.

[12] Malone K, Croke J, Malone C, Malone S (2012). Successful salvage using combined radiation and ABMT for patients with recurrent CNS NGGCT following failed initial transplant. BMJ Case Rep.

[13] Kellie SJ, Boyce H, Dunkel IJ, Diez B, Rosenblum M, Brualdi L, Finlay JL (2004). Primary chemotherapy for intracranial nongerminomatous germ cell tumors: results of the second international CNS germ cell study group protocol. J Clin Oncol.

[14] Kellie SJ, Boyce H, Dunkel IJ, Diez B, Rosenblum M, Brualdi L, Finlay JL (2004). Intensive cisplatin and cyclophosphamide-based chemotherapy without radiotherapy for intracranial germinomas: failure of a primary chemotherapy approach. Pediatr Blood Cancer.

[15] da Silva NS, Cappellano AM, Diez B, Cavalheiro S, Gardner S, Wisoff J, Kellie S, Parker R, Garvin J, Finlay J (2010). Primary chemotherapy for intracranial germ cell tumors: results of the third international CNS germ cell tumor study. Pediatr Blood Cancer.

[16] Nguyen QN, Chang EL, Allen PK, Maor MH, Ater JL, Mahajan A, Wolff JE, Weinberg JS, Woo SY (2006). Focal and craniospinal irradiation for patients with intracranial germinoma and patterns of failure. Cancer.

[17] Bouffet E (2010). The role of myeloablative chemotherapy with autologous hematopoietic cell rescue in central nervous system germ cell tumors. Pediatr Blood Cancer.

[18] Merchant TE, Boop FA, Kun LE, Sanford RA (2008). A retrospective study of surgery and reirradiation for recurrent ependymoma. Int J Radiat Oncol Biol Phys.

[19] Wetmore C, Herington D, Lin T, Onar-Thomas A, Gajjar A, Merchant TE (2014). Reirradiation of recurrent medulloblastoma: does clinical benefit outweigh risk for toxicity?. Cancer.

[20] Stafford SL, Pollock BE, Foote RL, Gorman DA, Nelson DF, Schomberg PJ (2000). Stereotactic radiosurgery for recurrent ependymoma. Cancer.

[21] Lo SS, Abdulrahman R, Desrosiers PM, Fakiris AJ, Witt TC, Worth RM, Dittmer PH, Desrosiers CM, Frost S, Timmerman RD (2006). The role of Gamma Knife Radiosurgery in the management of unresectable gross disease or gross residual disease after surgery in ependymoma. J Neurooncol.

[22] Stauder MC, Ni Laack N, Ahmed KA, Link MJ, Schomberg PJ, Pollock BE (2012). Stereotactic radiosurgery for patients with recurrent intracranial ependymomas. J Neurooncol.

[23] Bakst RL, Dunkel IJ, Gilheeney S, Khakoo Y, Becher O, Souweidane MM, Wolden SL (2011). Reirradiation for recurrent medulloblastoma. Cancer.

[24] Bouffet E, Hawkins CE, Ballourah W, Taylor MD, Bartels UK, Schoenhoff N, Tsangaris E, Huang A, Kulkarni A, Mabbot DJ, Laperriere N, Tabori U (2012). Survival benefit for pediatric patients with recurrent ependymoma treated with reirradiation. Int J Radiat Oncol Biol Phys.

[25] Nanda R, Dhabbaan A, Janss A, Shu HK, Esiashvili N (2014). The feasibility of frameless stereotactic radiosurgery in the management of pediatric central nervous system tumors. J Neurooncol.

[26] Liu SH, Murovic J, Wallach J, Cui G, Soltys SG, Gibbs IC, Chang SD (2016). CyberKnife radiosurgery for brainstem metastases: Management and outcomes and a review of the literature. J Clin Neurosci.

[27] Seymour ZA, Fogh SE, Westcott SK, Braunstein S, Larson DA, Barani IJ, Nakamura J, Sneed PK (2015). Interval from imaging to treatment delivery in the radiation surgery age: How long is too long?. Int J Radiat Oncol Biol Phys.

[28] Lightstone AW, Benedict SH, Bova FJ, Solberg TD, Stern RL; American Association of Physicists in Medicine Radiation Therapy Committee (2005). Intracranial stereotactic positioning systems: Report of the American Association of Physicists in Medicine Radiation Therapy Committee Task Group no. 68. Med Phys.

[29] Li G, Ballangrud A, Chan M, Ma R, Beal K, Yamada Y, Chan T, Lee J, Parhar P, Mechalakos J, Hunt M (2015). Clinical experience with two frameless stereotactic radiosurgery (fSRS) systems using optical surface imaging for motion monitoring. J Appl Clin Med Phys.

[30] Tryggestad E, Christian M, Ford E, Kut C, Le Y, Sanguineti G, Song DY, Kleinberg L (2011). Inter- and intrafraction patient positioning uncertainties for intracranial radiotherapy: a study of four frameless, thermoplastic mask-based immobilization strategies using daily cone-beam CT. Int J Radiat Oncol Biol Phys.

[31] Ramakrishna N, Rosca F, Friesen S, Tezcanli E, Zygmanszki P, Hacker F (2010). A clinical comparison of patient setup and intra-fraction motion using frame-based radiosurgery versus a frameless image-guided radiosurgery system for intracranial lesions. Radiother Oncol.

[32] Wong K, Cheng J, Bowlin K, Olch A (2016). Adaptation of vacuum-assisted mouthpiece head immobilization system for precision infant brain radiation therapy. Pract Radiat Oncol.

[33] Yang JC, Terezakis SA, Dunkel IJ, Gilheeney SW, Wolden SL (2016). Intensity-modulated radiation therapy with dose painting: A brain-sparing technique for intracranial germ cell tumors. Pediatr Blood Cancer.

[34] Mayo CS, Ding L, Addesa A, Kadish S, Fitzgerald TJ, Moser R (2010). Initial experience with volumetric IMRT (RapidArc) for intracranial stereotactic radiosurgery. Int J Radiat Oncol Biol Phys.

[35] Lu XQ, Mahadevan A, Mathiowitz G, Lin PJ, Thomas A, Kasper EM, Floyd SR, Holupka E, La Rosa S, Wang F, Stevenson MA (2012). Frameless angiogram-based stereotactic radiosurgery for treatment of arteriovenous malformations. Int J Radiat Oncol Biol Phys.

[36] Hu YW, Huang PI, Wong TT, Ho DM, Chang KP, Guo WY, Chang FC, Shiau CY, Liang ML, Lee YY, Chen HH, Yen SH, Chen YW (2012). Salvage treatment for recurrent intracranial germinoma after reduced-volume radiotherapy: a single-institution experience and review of the literature. Int J Radiat Oncol Biol Phys.

[37] Lawson JD, Wang JZ, Nath SK, Rice R, Pawlicki T, Mundt AJ, Murphy K (2010). Intracranial application of IMRT based radiosurgery to treat multiple or large irregular lesions and verification of infra-red frameless localization system. J Neurooncol.

[38] Sharma SD, Jalali R, Phurailatpam RD, Gupta T (2009). Does intensity-modulated stereotactic radiotherapy achieve superior target conformity than conventional stereotactic radiotherapy in different intracranial tumours?. Clin Oncol (R Coll Radiol).

[39] Schlesinger D, Xu Z, Taylor F, Yen CP, Sheehan J (2012). Interfraction and intrafraction performance of the Gamma Knife Extend system for patient positioning and immobilization. J Neurosurg.

[40] McTyre E, Helis CA, Farris M, Wilkins L, Sloan D, Hinson WH, Bourland JD, Dezarn WA, Munley MT, Watabe K, Xing F, Laxton AW, Tatter SB, Chan MD (2016). Emerging indications for fractionated Gamma Knife radiosurgery. Neurosurgery.

[41] Pan H, Cerviño LI, Pawlicki T, Jiang SB, Alksne J, Detorie N, Russell M, Carter BS, Murphy KT, Mundt AJ, Chen C, Lawson JD (2012). Frameless, real-time, surface imaging-guided radiosurgery: clinical outcomes for brain metastases. Neurosurgery.

[42] Li G, Ballangrud A, Kuo LC, Kang H, Kirov A, Lovelock M, Yamada Y, Mechalakos J, Amols H (2011). Motion monitoring for cranial frameless stereotactic radiosurgery using video-based three-dimensional optical surface imaging. Med Phys.

[43] Mancosu P, Fogliata A, Stravato A, Tomatis S, Cozzi L, Scorsetti M (2016). Accuracy evaluation of the optical surface monitoring system on EDGE linear accelerator in a phantom study. Med Dosim.

[44] Wen N, Li H, Song K, Chin-Snyder K, Qin Y, Kim J, Bellon M, Gulam M, Gardner S, Doemer A, Devpura S, Gordon J, Chetty I, Siddiqui F, Ajlouni M, Pompa R, Hammoud Z, Simoff M, Kalkanis S, Movsas B, Siddiqui MS (2015). Characteristics of a novel treatment system for linear accelerator-based stereotactic radiosurgery. J Appl Clin Med Phys.

[45] Seravalli E, van Haaren PMA, van der Toorn PP, Hurkmans CW (2015). A comprehensive evaluation of treatment accuracy, including end-to-end tests and clinical data, applied to intracranial stereotactic radiotherapy. Radiother Oncol.

[46] Merchant TE, Davis BJ, Sheldon JM, Leibel SA (1998). Radiation therapy for relapsed CNS germinoma after primary chemotherapy. J Clin Oncol.

[47] Sharma MS, Kondziolka D, Khan A, Kano H, Niranjan A, Flickinger JC, Lunsford LD (2008). Radiation tolerance limits of the brainstem. Neurosurgery.

[48] Xue J, Goldman HW, Grimm J, LaCouture T, Chen Y, Hughes L, Yorke E (2012). Dose-volume effects on brainstem dose tolerance in radiosurgery. J Neurosurg.

[49] Peng LC, Kahler D, Samant S, Li J, Amdur R, Palta JR, Liu C (2010). Quality assessment of frameless fractionated stereotactic radiotherapy using cone beam computed tomography. Int J Radiat Oncol Biol Phys.

[50] Keshavarzi S, Meltzer H, Ben-Haim S, Newman CB, Lawson JD, Levy ML, Murphy K (2009). Initial clinical experience with frameless optically guided stereotactic radiosurgery/radiotherapy in pediatric patients. Childs Nerv Syst.

